# Formulation and development of Serratiopeptidase enteric coated tablets and analytical method validation by UV Spectroscopy

**DOI:** 10.1155/2021/9749474

**Published:** 2021-10-19

**Authors:** Vijay Kumar Panthi, Saurav Kumar Jha, Raghvendra Chaubey, Rudra Pangeni

**Affiliations:** ^1^Department of Pharmacy, Tribhuvan University, Sunsari Technical College, Dharan, Sunsari, Nepal; ^2^Research & Development Department, Asian Pharmaceuticals, Rupandehi, Nepal; ^3^Research & Development Department, Corel Pharmaceuticals, Rupandehi, Nepal; ^4^Himalayan Parenteral and Formulations, Janakpur, Nepal; ^5^Quality Assurance Department, Asian Pharmaceuticals, Rupandehi, Nepal; ^6^Department of Pharmaceutical Sciences, School of Health and Allied Sciences, Pokhara University, Pokhara, Nepal

## Abstract

Serratiopeptidase (SRP) is a proteolytic enzyme that emerged as one of the most potent anti-inflammatory and analgesic drugs. The purpose of the present study was to formulate and evaluate enteric-coated tablets for SRP and investigate their stability using a simple and validated analytical method by ultraviolet (UV) spectroscopy. The colloidal silicon dioxide (2.50%), sodium starch glycolate (3.44%), and crospovidone (2.50%) were used as appropriate excipients for the development of core part of tablets. To protect the prepared tablets from acidic environment in the stomach, white shellac, castor oil, HPMC phthalate 40, and ethyl cellulose were used. The seal coating and enteric coating attained were 2.75% and 6.74%, respectively. SRP was found to be linear at 265 nm in the concentration range of 25–150 *µ*g/mL. The results revealed that our developed method was linear (*R*^2^ = 0.999), precise (RSD % = 0.133), and accurate (% recovery = 99.96–103.34). The formulated SRP tablets were found to be stable under accelerated conditions as well as under room temperature for 6 months (assay %: >97.5%). The *in vitro* drug release study demonstrated that enteric-coated tablets were able to restrict SRP release in both acidic environments: 0.1 N HCl and simulated gastric fluid (pH 1.2). Moreover, at 60 minutes, the formulated SRP tablets revealed 13.0% and 8.98% higher drug release in phosphate buffer (pH 6.8) and simulated intestinal fluid (pH 6.8), respectively, compared to the marketed tablet formulation. This study concludes that enteric-coated tablets of SRP with higher drug release in the intestine can be prepared and examined for their stability using validated analytical technique of UV spectroscopy.

## 1. Introduction

A wide range of proteolytic enzymes mixtures have been used as supportive therapy in clinical condition such as trauma and orthopedic. There are several reports supporting the use of these proteolytic enzymes for the treatment of inflammatory disorders [1]. Moreover, these enzymes linked to metalloprotease family have been profitably assessed for their anti-inflammatory properties, which include trypsin, chymotrypsin, and serratiopeptidase (SRP) [2]. Chemically, SRP is an extracellular metalloprotease derived from the nonpathogenic enterobacteria *Serratia* E15 [3]. It consists of a polypeptide chain of 470 residues and a catalytic zinc ion per molecule with molecular weight of 45–60 kDa. SRP is normally recommended for oral administration at a dose of 5–10 mg three times a day [1]. This enzyme is rapidly and readily absorbed through the intestine and transported directly into the blood circulation [4]. SRP has been extensively used in Europe and Asia for more than 30 years but is relatively new in the United States and Canada. In addition, its anti-inflammatory property was first studied in Japan in 1967. Later, during the 1970s, these parenteral enzyme preparations were replaced by oral enteric-coated tablet form. During the 1980s and 1990s, this research was proposed to separately be conducted in Europe and Japan and demonstrated that SRP is the most effective agent in diminishing inflammation among all available enzyme formulations [3, 5]. Currently, SRP has been broadly used in Japan and Europe as the anti-inflammatory and analgesic agent of choice [6].

The most common problem associated with SRP oral delivery is high risk of enzymatic degradation in the gastrointestinal tract due to its proteinaceous nature. In addition, hydrophilic nature of SRP leads to low intestinal membrane permeability, due to which a very high dosage of SRP needs to be orally administered to elicit significant anti-inflammatory responses [7]. In order to increase the stability of SRP (reduced acid hydrolysis) and improve the oral bioavailability simultaneously, several techniques like delivering the SRP entrapped in Eudragit S100 microspheres, liposomal formulations, alginate gel encapsulated with SRP, chitosan-coated ceramic nanocores containing SRP, in situ cubic phase transforming system of glyceryl monooleate containing SRP, tetracycline-SRP-containing periodontal gel, and polar lipid-based lipospheres were approached previously [1]. Oral tablet dosage form with enteric coating and combination with controlled and sustained release property could be an effective way to decrease the frequency of dosing and increase the bioavailability of SRP [8–10]. Therefore, *in vitro* release profiles and *in vivo* efficacy are considered as important parameters to be studied for developing suitable delivery system. Another major challenge in developing a formulation of SRP is to retain its structural backbone, inhibiting the folding tendency during manufacturing, storage, digestion, and absorption in the stomach and intestines [11].

In order to determine the qualitative and quantitative evaluation of SRP, different analytical techniques have been advised including thin layer chromatography (TLC), X-ray powder diffractometry (XRPD), enzyme-linked immunosorbent assay (ELISA), high-performance liquid chromatography (HPLC), and ultraviolet (UV) spectroscopy. Meanwhile, some drawbacks still exist behind the successful application of these techniques. Although TLC technique is easy and fast for the qualitative measurement and identification of SRP, it has demerits like long plate running time and laboriousness. Furthermore, XRPD is very expensive, and the cost involved in purchase of the instrument and its upkeep may prevent its use for estimation of SRP is a simple routine tool. Moreover, although ELISA appears to be simple, it is very difficult to receive the antibodies against SRP. The cost involved to carry out a single analysis is a factor for the technique being widely used. In addition, requirement of specific column and solvents may limit the adequate utilization of HPLC for SRP determination. On the contrary, many studies revealed that UV spectrophotometric method was found to be precise, accurate, robust, simple, and economical for the determination of SRP alone and in combination with other drugs including aceclofenac, diclofenac, and nimesulide [3]. In addition, one of the advantages of the application of UV method over other analytical techniques is that the UV method does not require time consuming treatments and procedures usually associated with chromatographic methods as well as rapid analytical techniques in the early phase of product development [12, 13]. The main objective of this research was to formulate stable enteric-coated tablets of SRP to prevent the degradation in gastric environment and develop and validate simple and cost-effective UV spectroscopy method for the determination of SRP.

## 2. Materials and Methods

### 2.1. Materials

Serratiopeptidase (SRP) powder was purchased from Biocon Limited, Bangalore, India. Isopropyl alcohol and methylene chloride were procured from Thermo Fisher GmbH, Karlsruhe, Germany. Microcrystalline cellulose powder (MCCP) pH 101 and 102, colloidal silicon dioxide, crospovidone, magnesium stearate, and stearic acid were purchased from Thermax Pvt. Ltd., India. Hydroxypropyl methyl cellulose (HPMC) phthalate 40 was obtained from Shandong Head Co., Ltd., China. The other coating materials were obtained as a gift sample from Colorcon Asia Pvt. Ltd, Industrial Estate, Goa, India. Marketed enteric-coated tablet formulation of SRP was procured from local medical store and used as a reference sample. All the reagents and solvents used in the analysis were of analytical grade.

### 2.2. Instrumentation

The following instruments were used in the formulation and analysis of prepared enteric-coated tablets: dissolution study was performed using dissolution tester (Electrolab, model: EDT-14XL), UV/visible spectrophotometer (Agilent Technologies, model: Cary-60 UV-Vis), tray dryer (Techmac Engineering Works, Azadpur, Delhi-33, India), compression machine (Fluidpack, Machine No. 145, Ahmedabad, India), analytical balance (Ohaus Corporation, model: PA423C, USA), thickness and diameter (Electronic Express, model: 0604CAL6++), hardness tester (Electrolab, EH-01P), and coating machine (Captech Systems, model: CCP-12).

### 2.3. Analytical Method Development

#### 2.3.1. Determination of Wavelength of Maximum Absorption of SRP

The 25–150 *µ*g/mL of concentration range of SRP was dissolved in deionized (DI) water and the UV-visible spectrum of SRP was examined using UV spectrophotometer between 200 and 400 nm. Next, the derivative spectra of SRP at various concentrations were recorded. The interfering effect of all excipients on the maximum absorption was assessed by scanning the spectrum of each excipient alone and in combination with SRP. The maximum absorbance was observed at 265 nm.

#### 2.3.2. Preparation of Standard Stock Solution of SRP

Standard solution was prepared by weighing 50 mg of SRP accurately and then was transferred to a 100 mL volumetric flask and dissolved by adding 25 mL of DI water. The flask was allowed to sonicate for 15 minutes and volume was made up to the mark with DI water to give a solution containing 500 *µ*g/mL of SRP.

#### 2.3.3. Determination of Calibration Curve of SRP

The required volume from the standard stock solution of SRP was withdrawn and transferred to a 10 mL volumetric flask. The final volume was adjusted up to the mark with DI water to obtain different concentration of SRP including 25, 50, 75, 100, 125, and 150 *µ*g/mL. The calibration curve of concentration versus absorbance was plotted and then straight-line equation was determined.

#### 2.3.4. Analysis of Formulated and Commercial Tablet Formulation

The developed method was also applied to determine SRP in formulated and marketed tablets. An average weight of SRP tablet equivalent to 50 mg was taken in 50 mL volumetric flask and volume was made up to the mark with diluents. From this, 5 mL was taken and transferred to a 50 mL volumetric flask and the final volume was made up to the mark with diluent to give a final concentration of 100 *µ*g/mL. The absorption was recorded at 265 nm and the concentrations of the drug were calculated from the linear regression equation. The percentage assay and relative standard deviation (RSD %) of the SRP in tablets were calculated in triplicates.

### 2.4. Analytical Method Validation

#### 2.4.1. Linearity

The linearity and range of the developed method were analyzed by measuring the absorption of a series of SRP standard solutions (25–150 *µ*g/mL) at *λ*_max_ of 265 nm. The absorption of these standard solutions was plotted against different concentration. The regression line equation and the square of the correlation coefficient (*R*^2^) were calculated.

#### 2.4.2. Accuracy

In this research, the accuracy of the method was determined by addition of standard drug to the sample at three different concentration levels (50, 100, and 150%) taking into consideration percentage purity of added bulk drug samples. It was determined by calculating the recovery of SRP by standard addition method. In addition, accuracy of the developed method in SRP enteric-coated tablet formulation was studied by the analysis of spiked samples at different concentrations as mentioned above.

#### 2.4.3. Precision

Precision studies were performed by analyzing standard solutions containing 50, 100, and 150 *µ*g/mL on three different days by two different analysts as an interday precision. The absorbance of the solutions was examined and then standard deviation (SD) and RSD % were calculated. Moreover, to determine intraday precision, standard solutions containing 50, 100, and 150 *µ*g/mL of SRP were analyzed three times on the same day. Next, absorbance of the solutions was examined and standard deviation (SD) and RSD % were calculated.

#### 2.4.4. Limit of Detection (LOD) and Limit of Quantitation (LOQ)

The calibration curve was repeated for 6 times and SD of the intercepts was calculated using the following formula:

(1) $$\begin{array}{c} \text{LOD}=\frac{\left(3.3*\text{SD}\right)}{\text{Slope}}, \end{array}$$

(2) $$\begin{array}{c} \text{LOQ}=\frac{\left(10*\text{SD}\right)}{\text{Slope}}, \end{array}$$

  where SD is standard deviation of Y- intercept of 6 calibration curves.
  Furthermore, SD intercept = SE intercept *∗*√N.
 
*N* is number of test samples.
  Slope is the mean slope of the 6 calibration curves [14, 15].

#### 2.4.5. Robustness

The robustness of the method was evaluated by examining the different mixture of same concentration of samples to find out the effect of slight changes on absorption at wavelengths of 265 ± 1.0, and then average mean, SD, and RSD % of absorbance were determined.

### 2.5. Formulation and Development of SRP Tablets

The core part of SRP tablets was prepared by dry mixing process followed by slugging using roller compactor prior to improving the powder flow properties of granules. This process was performed without applying heat to avoid thermal denaturation. Slugging is a dry granulation that uses mechanical compression (slugs) or compaction (roller compaction) to facilitate the agglomeration of dry powder particles. The conformational changes in proteins that occur when they are heated are referred to as denaturation [16, 17]. Before commencement of manufacturing process, MCCP pH 101 and 102 and crospovidone were allowed to dry at 100°C for 30 minutes. After drying, MCCP pH 101 and 102 and crospovidone were sieved through 30 meshes and half quantities of three excipients (colloidal silicon dioxide, purified talc, and stearic acid) were sieved through 60 meshes and mixed properly for 10 minutes. Subsequently, mixed powder was sieved through roller compactor as a slugging process and materials were passed through 20 meshes. Finally, remaining amounts of colloidal silicon dioxide, purified talc, and stearic acid were sifted through 60 meshes for lubrication. Consequently, lubricated granules were compressed in 8-station rotary compression machine using dies and punch having 7.4 mm diameter without break line.

**Figure 1:**
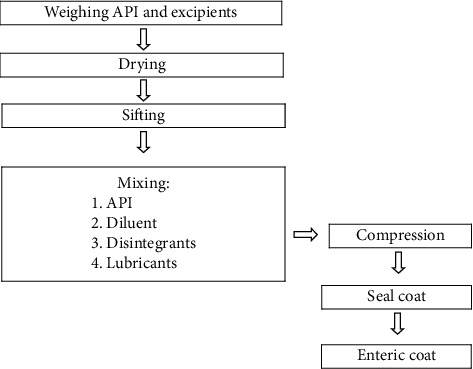
Manufacturing procedure of SRP tablets.

To seal-coat the tablet, white shellac was dissolved in isopropyl alcohol (IPA) with continuous stirring for about 30 minutes and then castor oil was added in it and it was allowed to stir for 10 minutes. For preparation of enteric coating solution, HPMC phthalate 40 was dispersed in IPA with continuous stirring for 10 minutes and then methylene chloride was added in it followed by stirring continuously for 30 minutes prior to dissolving. Subsequently, ethyl cellulose (EC), castor oil, titanium dioxide, and Lake Erythrosine were added in this solution with regular stirring approximately for 30 minutes. In this study, coating of tablets was done using a side-vented, perforated pan coating apparatus machine. Firstly, weighed quantity (about 1.0 kg) of tablets was kept in the coating pan which was preadjusted at 40°C for 5–10 minutes and actual weight of tablet was evaluated. Secondly, tube was put in the coating solution, and then the various parameters including spray rate (8–20 mL/min), inlet air temperature (20–50°C), atomizing air pressure (1–3 bar), and the rotating speed of coating pan (8–15 rpm) were adjusted and optimized. Furthermore, after completion of coating, tablets were kept in the pan at 37°C and 3 rpm for curing. Finally, coated tablets were taken from the pan and the various parameters were assessed. Although EC is not an enteric coating material, this excipient was used in this study owing to its binding properties to enhance the hardness of tablet as well as to avoid drug release in gastric region. EC is water-insoluble cellulose having various appealing characteristics including biocompatibility, gastroresistance, and degradation to nontoxic and readily excreted components. This material is widely applied in pharmaceutical technology because it is convenient for processing alone or in combination with other excipients such as plasticizers or other polymers [18]. Furthermore, due to the potentiality of EC to modify release rate of drug, it is extensively utilized as a coating agent. A study done by Shah et al. developed a colon targeted multiunit formulation of metronidazole using EC and Eudragit^®^ S 100 as coating polymers to prevent initial drug release in the gastric region. The drug release was remarkably hindered by the outer EC-based coating layer [19]. Moreover, EC, on the other hand, has ethoxy functional groups with a very low propensity to react chemically. This polymer, then, could provide the formulator good binding characteristics with low chemical reactivity [20]. The detailed composition of the tablet formulation is mentioned in Table 1 and the overall procedure was illustrated in flowchart shown in Figure 1.

### 2.6. Loss on Drying (LOD) of Granules

After successful drying, approximately 5 gm sample was taken from tray dryer and poured in moisture analyzer (Sartorius MA37) containing weighing pan and distributed over its surface area and finally allowed to heat at 80°C. Then LOD value of dried granules was reported from moisture analyzer.

### 2.7. Carr's Index and Hausner Ratio of Lubricated Granules

After complete drying of moistened granules, lubricants were added and mixed properly for 5 minutes in order to enhance the flow properties of lubricated granules. The 10 gm sample of lubricated granules was poured in 100 mL measuring cylinder and tapped 100 times. Next, Carr's index and Hausner ratio of lubricated granules were determined. The ratio of tapped density to bulk density of the powders is called the Hausner ratio [21].

### 2.8. Weight Variation and Physical Specification

Weight variation of the formulated tablets was evaluated according to USP method recommended for uncoated tablets. This parameter is performed by weighing 20 tablets separately and if it satisfies the criteria approved by official pharmacopoeia, test will be considered successful [15]. Tablet physical specifications such as hardness were evaluated by digital portable hardness tester (Electrolab, Model EH-01P), while thickness and diameter were examined by Vernier caliper (Electronic Express, model: 0604CAL6++). The test was performed on ten tablets; the average reading was assigned as the tablet hardness, thickness, and diameter specifications of the formulated tablets. In addition, for appearance examination, twenty tablets of each formulation were randomly selected to check any discoloration or degradation of drug in the tablets by visual method. If any discoloration or black spots appear, it shows the degradation or decomposition of drug in the tablet formulation [22].

### 2.9. Determination of Content Uniformity

According to European Pharmacopeia (Ph. Eur.), for the assessment of content uniformity, 10 tablets were subdivided and ten different subdivided parts were randomly selected. The active content in each part was determined using a suitable analytical method. Ten tablets were finely powdered; amount of the powder equivalent to 10.0 mg of SRP was accurately weighed and transferred to a 100 mL of volumetric flask. Subsequently, the flask was filled with phosphate buffer pH 6.8 and mixed thoroughly. The volume make-up was done up to the mark with the diluent to give a final concentration of 100 *µ*g/mL and filtered. Moreover, the absorbance of the resulting solution was measured at 265.0 nm using UV spectroscopy. Finally, the linearity equation obtained from calibration curve as mentioned previously was used for the estimation of SRP in the tablet formulations. The preparation complies with the test if each individual content is between 85.0% and 115.0% of the average content. The preparation fails to comply with the test if more than one individual content is outside these limits or if one individual content is outside the limits of 75.0–125.0% of the average content. If one individual content is outside the limits of 85.0–115.0% but within the limits of 75.0–125.0%, the individual contents of 20 other units (subdivided tablet parts) taken at random are determined. The preparation complies with the test if not more than one of the individual contents of the 30 units is outside 85.0–115.0% of the average content and none are outside the limits of 75.0–125.0% of the average content [23].

### 2.10. *In Vitro* Dissolution Studies

#### 2.10.1. Drug Release Profile in 0.1 N HCl (pH 1.2) and Phosphate Buffer (pH 6.8)

The dissolution of formulated SRP enteric-coated tablets was examined as per the USP and International Conference on Harmonization (ICH) guidelines. This study was performed using USP apparatus 2 (paddle). The drug release study was carried out in two different dissolution media, 0.1 N HCl (pH 1.2) and phosphate buffer (pH 6.8), according to USP. After temperature reached 37 ± 0.5°C, single tablet was added individually to separate dissolution dishes containing 900 mL of dissolution medium and run for 60 minutes at 50 rpm. Subsequently, 5 mL of the solution was withdrawn using syringes from each dissolution container at predetermined time intervals of 15, 30, 45, and 60 minutes. After each withdrawal, 5 mL of fresh dissolution media was added to maintain sink condition. The mean value obtained from triplicate measurements was taken to assess the percentage of dissolved SRP using the following formula [24]:

(3) $$\begin{array}{c} \text{\% of dissolved SRP}=\frac{\text{actual amount of released SRP}}{\text{theoretical amount of SRP}}\times 100\text{.} \end{array}$$

The comparative evaluation of dissolution was established on the readings of the evaluated similarity factor (*f*2) and dissimilarity factor (*f*1) of the dissolution profile for the formulated and marketed SRP tablets. Moreover, the values for *f*1 and *f*2 were examined using (4) and (5), respectively. The *f*2 factor evaluates the vicinity between two release profiles and similarly *f*1 also calculates the difference between two profiles. *R*_t_ and *T*_t_ in the equations represent the percentages of drug dissolved at each time point for the reference (market tablet) and test (formulated tablet), respectively. The *f*1 value higher than 15 demonstrates significant dissimilarity, while the *f*2 value more than 50 reveals significant similarity [15, 24–26].

(4) $$\begin{array}{c} f_{1}=\left\{\frac{\left[\sum_{t=1}^{n}\left|R_{t}-T_{t}\right|\right]}{\left[\sum_{t=1}^{n}R_{t}\right]}\right\}\times 100, \end{array}$$

(5) $$\begin{array}{c} f_{2}=50\;\cdot \;\log\;{\left\{\left[1+\frac{1}{n}\overset{n}{\underset{t=1}{\sum }}\left(R_{t}-T_{t}\right)²\right]\right\}}^{5}\times 100. \end{array}$$

#### 2.10.2. Drug Release Profile in Simulated Gastric Fluid (pH 1.2) and Simulated Intestinal Fluid (pH 6.8)

The release assessment of SRP enteric-coated tablets applied a calibrated dissolution apparatus with paddles at 50 rpm, and the bath temperature was maintained at 37 ± 0.5°C. It is suggested that, for all the enteric-coated tablets, they must tolerate simulated gastric fluid (pH 1.2) in the initial 2h followed by 1 h in simulated intestinal fluid (pH 6.8) according to the standard method, since SRP will hydrolyze in aqueous solution. Therefore, in this study, we employed simulated intestinal fluid (pH 6.8) that consisted of 0.25% sodium dodecyl sulphate (SDS) as release medium. For preparation of simulated gastric fluid, NaCl (3 g) was dissolved in about 1450 mL of DI water with continuous stirring and then pH was adjusted to 1.2 ± 0.1 with diluted HCl. Furthermore, the simulated intestinal fluid was also composed by dissolving potassium phosphate monobasic (10.2 g) and SDS (3.75 g) in a same 1000 mL of DI water and then pH adjusted to 6.8 ± 0.1 with 1 N NaOH. Finally, the volume make-up of each prepared fluid was done up to 1500 mL with DI water [27].

- Tolerance test in simulated gastric fluid
- Tolerance test of SRP enteric-coated tablets was performed in 250 mL simulated gastric fluid (pH 1.2) with a paddle rotation of 50 rpm and with the bath temperature of 37°C. After 2 h, 5 mL sample was taken by syringes from each vessel and filtered through a nylon filter (0.45 m, 25 mm). After each withdrawal, same volume (5 mL) of fresh dissolution media was added to maintain sink condition. The mean value obtained from triplicate measurements was taken to assess the percentage of dissolved SRP using the formula mentioned in equation (1).
- Release profile in simulated intestinal fluid
- The release test of the enteric-coated tablets required enduring for 2 h in 250 mL of simulated gastric fluid, and the release amount was determined in 250 mL of the simulated intestinal fluid. Sample was withdrawn from each dissolution apparatus at 15, 30, 45, and 60 min during dissolution in simulated intestinal fluid, and the same amount of release medium was added promptly. Moreover, the withdrawn sample solutions were filtered through a nylon filter (0.45 m, 25 mm), and then the release percentage of SRP enteric-coated tablets in simulated intestinal fluid (pH 6.8) was determined and calculated according to the formula mentioned in equation (1).

### 2.11. Accelerated Stability Studies

Stability studies were carried out on optimized formulation according to ICH guidelines. The formulated tablet packed in blister strips was subjected to accelerated stability testing for 6 months as per ICH norms at a temperature of 40 ± 2°C and relative humidity of 75 ± 5%. Samples were taken at regular time intervals of 1 month for over a period of 6 months and analyzed for the change in color, hardness, drug content, and in vitro drug release in acid media (0.1 N HCl) by procedure stated earlier. Any changes in evaluation parameters, if observed, were noted [28]. Tests were carried out in triplicates and mean value of the observed values was noted along with standard deviation.

## 3. Statistical Analyses

Statistical analyses were performed with GraphPad Prism 5.0 (GraphPad Software, Inc., San Diego, CA). The data were evaluated using one-way analysis of variance (ANOVA) followed by Tukey's multiple comparison test. *P* < 0.05 was considered to indicate statistical significance. All results are expressed as mean ± standard deviation.

## 4. Results and Discussion

### 4.1. Analytical Method Validation

#### 4.1.1. Linearity

**Figure 2:**
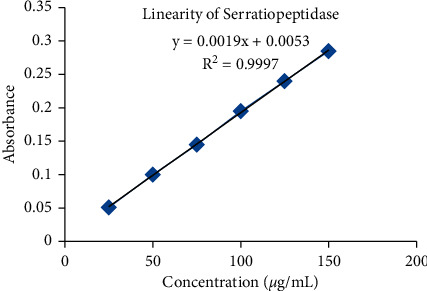
UV standard curve and linearity of SRP in the concentration range of 25–150 *µ*g/mL in water at 265 nm.

The SRP was found to be linear at 265 nm in the concentration range of 25–150 *µ*g/mL in water, which was demonstrated in calibration curve. The obtained correlation coefficient (*R*^2^) and regression equation were 0.999 and *Y* = 0.001*x* + 0.005, respectively. The linearity range of SRP is shown in Figure 2. Furthermore, Nirale and Menon in 2010 studied the stability of SRP over a period of 12 h in DIW and Tris and phosphate buffer at room temperature and 37°C. The study that measured enzyme activity using proteolytic activity measurement method demonstrated retention of SRP enzyme activity of higher than 90% in Tris and phosphate buffer at both temperatures for at least 8 h. However, the stability of SRP was significantly decreased in DIW [2].

#### 4.1.2. Accuracy

For accuracy determination, the mean recovery % (*n* = 3) of SRP were 99.9% (50% level), 100.3% (100% level), and 100.2% (150% level). Results demonstrate that the recovery of SRP in spikes standard solution at different concentration range was between 99.9 and 100.3% with lower relative standard deviation (≤2%), ensuring the accuracy of the established method (Table 2). In addition, spike recovery in prepared formulation at 50%, 100%, and 150% spike level with RSD percentage of 0.190% also demonstrated the accuracy of the developed method in the real samples. Next, spike of blank tablet matrix with three different concentrations of SRP of 25, 50, and 75 *µ*g/mL at 50%, 100%, and 150% of SRP spike level also demonstrated the percentage recovery of 98.0–101.0%, demonstrating the accuracy of the method as well as no interference of matrix (data not shown).

#### 4.1.3. Precision

In the intraday precision analysis (*n* = 3) of SRP, the observed RSD percentages were 0.14%, 0.16%, and 0.10% at 50, 100, and 150 *µ*g/mL, respectively (Table 3). For the intraday precision with analyst 1 (*n* = 3), the 50, 100, and 150 *μ*g/mL of SRP revealed 0.17, 0.11, and 0.08 RSD %, respectively (Table 4). Moreover, for the intraday precision with analyst 2 (*n* = 3), the 50, 100, and 150 *μ*g/mL demonstrated 0.15, 0.09, and 0.006 RSD %, respectively (Table 5). The RSD values lower than 2.0% ensure the precision of the method [29].

#### 4.1.4. Determination of LOD and LOQ

The sensitivity of method was assessed by determining LOD and LOQ according to the formula mentioned in Section 2.4.4. The LOD and LOQ of SRP were found to be 5.28 and 16.00 *µ*g/mL, respectively. The results obtained are presented in Table 6.

#### 4.1.5. Robustness

Despite the same concentration (100 *µ*g/mL) of SRP, the three different wavelengths showed robustness below 2.00 RSD %, which is within the acceptance criteria. The obtained results are illustrated in Table 7.

### 4.2. Tablet Formulation Development

#### 4.2.1. LOD of Granules

Among five formulations, *F*4 demonstrated low LOD (1.72), followed by *F*5. *F*1 and *F*3 revealed nearly same LOD. There were not any noticeable difficulties for the compression of tablets in each composition. The detailed value of LOD is shown in Table 8.

#### 4.2.2. Carr's Index and Hausner Ratio of Lubricated Granules

**Figure 3:**
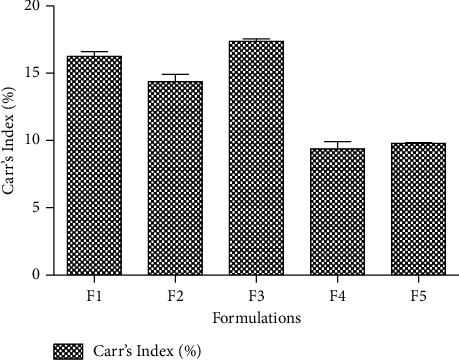
Carr's index (%) of different formulation of SRP. Values are expressed as mean ± SD (*n* = 3).

*F*4 and *F*5 showed Carr's index of 9.40 and 9.75, respectively, which means that powder flow was excellent as compared to other formulations (Figure 3). Moreover, *F*3 exhibited 17.2 (fair flow), followed by good flow properties of *F*1 and *F*2 [30]. The flow characteristics of granules were significantly improved after adding MCC pH 102 instead of MCC pH 101. Furthermore, *F*2 also showed Hausner ratio of 1.09, which illustrates its flow superiority as compared to other formulations (Table 8).

#### 4.2.3. Optimization of Formulation

**Figure 4:**
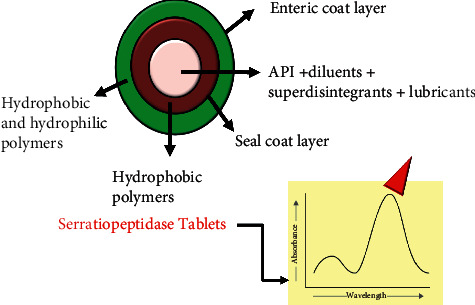
Design of SRP tablet and analyzed by UV spectroscopy.

All five formulations were compressed at same weight (160.00 mg) followed by seal coating and enteric coating. In formulation *F*1, the tablets were prepared using single superdisintegrants, that is, sodium starch glycolate (SSG), but disintegration time and the powder flow from hopper were not satisfactory, while rest of the compression parameters were appropriately good. In *F*2, the ratio of disintegrant was increased and MCCP pH 102 was included in the formulation instead of MCCP pH 101 to overcome the previous issue. The flow properties of granules were improved and tablet was completely disintegrated within 10 minutes. However, tablet appearance was not smooth. Furthermore, glidant, disintegrant, and lubricant proportion were increased in *F*3 along with another disintegrant (crospovidone). Although each tablet parameter was in the desired range and *F*3 was shifted for coating, formulation *F*3 demonstrated drug release in acid media. Moreover, concentration of white shellac was increased in *F*4 but seal coating layer was not sufficiently attached and started to crack. To address coating issue in previous approach, the ratio of white shellac, ethyl cellulose, and castor oil was added more in *F*5 (Table 1). The SRP tablets attained 2.75% seat coat and weight gain was found to be in the range of 3.95 to 4.45 mg. Core tablets containing 2.75% were fully coated with barrier coating without any kind of coating defect. So, this proportion of coating on core tablet was optimized concentration of seal coating [30, 31]. Moreover, tablets attained 6.74% as enteric coating and weight gain was observed to be between 9.82 and 10.48 mg. This ratio in *F*5 gave sufficient protection of core tablet in 0.1 N HCl and did not release approximately more than 1.50% drug. So, enteric coating formula of *F*5 was optimized for further study. The final appearance of tablets was pink colored, round, biconvex, and enteric-coated (Figure 4). Thus, only *F*5 was preceded further including detailed examination of tablets from day 1 to 6 months as accelerated analysis.

#### 4.2.4. Weight Variation and Physical Specification

Some of the physical parameters of formulated tablet including appearance, weight, hardness, thickness, and diameter were determined as tablet specification. The average value of the tested parameters will be considered as tablet specification. The physical appearance and hardness of formulated tablet were observed for 6 months at both 25° and 40°C (with 75% humidity). The detailed data of the tested parameters are shown in Tables 9 and 10.

From the evaluation of weight variation, the observed average, minimum, and maximum weight of formulated SRP were 172.77, 171.50, and 178.10 mg, respectively, which were within the limit of 170.00 mg ± 7.50%. The average thickness and diameter were 3.97 and 7.42 mm, respectively (Table 9). Furthermore, no noticeable physical change of tablet was observed till 6 months of stability for both room temperature and accelerated stability. In addition, during day 1, hardness of formulated tablets was 23.02 and 22.87 kgf at 25° and 40° (with 75% humidity), respectively, and until 6 months this parameter is not significantly declined as compared to day 1 (*P* > 0.05). Therefore, unchanged color of tablets and insignificant decline of hardness revealed that the formulated SRP tablet was physically stable until 6 months of stability in accelerated conditions. The overall findings are revealed in Table 10. Moreover, thickness of tablets was observed to be 3.94 ± 0.011 mm in 6 months' analysis of accelerated stability which was very close to initial measurements which further signified that formulated tablets were not gaining the moisture because tablets were not swelling during the stability period. In addition, crushed tablets demonstrated LOD 1.82 which was also almost same as original value. These findings exhibited that the hydrolysis problem may not appear in formulated tablets. Furthermore, to avoid the chances of hydrolysis, the tablets were prepared by dry granulation followed by alcoholic coating and packed in blister packaging. For products which are susceptible to hydrolysis, packaging may deliver an appropriate alternative for enhancing the stability of product. Previous study suggested that many blister packaging materials that were proper for moisture were poor for oxygen. Due to the higher surface area of a blister package per dose, permeation of moisture will be considered an issue under challenging conditions with all the blisters except foil [32]. However, still few studies are necessary for the confirmation of hydrolysis.

#### 4.2.5. Content Uniformity

**Figure 5:**
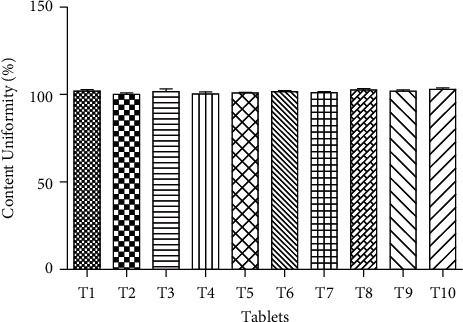
Content uniformity (%) of different formulation of SRP.

The uniformity content test was also performed according to the Ph. Eur. The results showed that no tablet assay % was out of the limit (85–115%) [33]. The determined content uniformity % of 10 tablets is presented in Figure 5.

#### 4.2.6. Dissolution Profile

**Figure 6:**
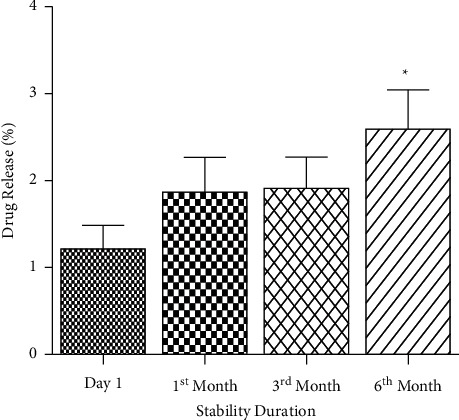
SRP release in acidic media (0.1 N HCl): ^*∗*^*P* < 0.05 compared to day 1.

**Figure 7:**
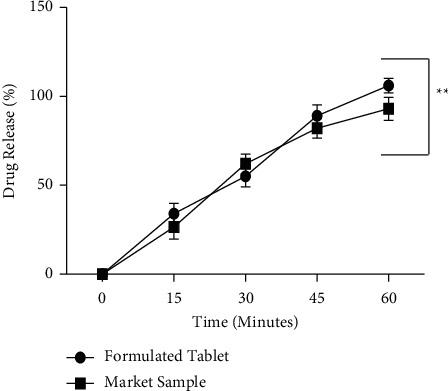
SRP release in buffer media (pH 6.8 phosphate buffer): ^∗∗^*P* > 0.05 compared to market sample.

Drug release percentage in 0.1 N HCl was measured in different stability schedules including day 1, 1st month, 3rd month, and 6th month as accelerated stability analysis and its release % were compared with day 1. The ranges of dissolution in acid media were 0.85–1.59% (day 1), 1.45–2.47% (1st month), 1.56–2.55% (3rd month), and 1.95–3.34% (6th month). In acid media, from day 1 to 6 months of stability, SRP demonstrated the release profile within the limit of no more than (NMT) 15.00%. No significant difference (*P* > 0.05) in acid uptake was observed until the 3rd month, while in 6th month the acid uptake % was remarkably higher (*P* < 0.05) as compared to day 1 which is considered as statistically significant, meaning that acid release was enhancing periodically but slowly (Figure 6). Moreover, acid release was significantly higher in 6 months of accelerated stability but the overall release value was sufficiently within the limit (NMT 15%) which can be considered as a gradual degradation. The data observed in acid release demonstrated that the formulated SRP tablet was physically stable until the 6th month in accelerated stability. In addition, drug release in acidic medium becomes higher with lapses of time when stored under accelerated condition as revealed in Figure 6. This may be due to the weakening of polymer matrix present in the coating part of tablets as evidenced by the decline in hardness during stability studies as shown in Table 10. Although such changes are increased periodically, they can be considered as acceptable because observed values were within the range [34]. Furthermore, release rate of SRP formulated tablet in pH 6.8 phosphate buffer was compared with market sample excluding accelerated analysis. Both formulations revealed remarkable release profile until 60 minutes. The corresponding figures of formulated tablet and market sample were 106.00% and 93.00%, respectively (Figure 7). However, the dissolution profile of the SRP formulated tablet revealed a slight dissolution improvement (13.00% higher) over the marketed SRP tablet. Thus, the dissolution findings exhibited that there was no statistical significance (*P* > 0.05) from 15 to 60 minutes between the formulated tablet and the marketed one.

**Figure 8:**
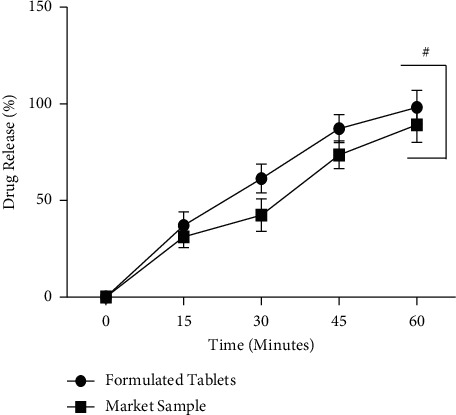
SRP release in simulated intestinal fluid (pH 6.8): ^#^*P* > 0.05 compared to market sample.

In addition, *in vitro* release profile of SRP from enteric-coated tablets was carried out in simulated gastric fluid (pH 1.2) for 2 h and findings are revealed in Table 11. The percentage of the residual content of SRP in enteric-coated tablets was determined in accordance with its initial amount. The results demonstrated that little amount of SRP was released from formulated enteric-coated tablets within 2 h of agitation. However, percentage of SRP released in simulated gastric fluid (pH 1.2) was 1.023-fold lower than market sample. Furthermore, no evidence of disaggregation, cracking, or melting was observed at the surface of enteric-coated tablets in simulated gastric fluid solution. Moreover, release profile of SRP from enteric-coated formulation and market sample over the period of 60 minutes in simulated intestinal fluid (pH 6.8) is revealed in Figure 8. Both samples showed significant release profile until 60 minutes. At 60 min, the formulated SRP tablets and market sample demonstrated 98.22% and 89.24% of drug release, respectively. In this medium, SRP tablets showed 8.98% higher dissolution as compared to market sample at 60 minutes. Interestingly, there was no significant variation in release profile of both formulated SRP and market sample in both phosphate buffer (pH 6.8) and simulated intestinal fluid (pH 6.8). Thus, the dissolution data showed that there was no statistical significance of drug release (*P* > 0.05) from 15 to 60 minutes between the formulated tablets and the marketed one in simulated intestinal fluid (pH 6.8) medium. Moreover, in this study, similarity factor (*f*2) and dissimilarity factor (*f*1) of optimized SRP enteric-coated tablets and market sample were determined in both phosphate buffer (pH 6.8) and simulated intestinal fluid (pH 6.8). The results of similarity factor (*f*2) applied in phosphate buffer (pH 6.8) and simulated intestinal fluid (pH 6.8) were 51.14 and 54.22, respectively. Therefore, it was concluded that optimized formulation of SRP enteric-coated tablets has shown good similarity (more than 50) with market sample. Furthermore, findings of dissimilarity factor (*f*1) in phosphate buffer (pH 6.8) and simulated intestinal fluid (pH 6.8) were 16.61 and 16.67, respectively. Thus, it was also further justified that an *f*1 value greater than 15 indicates significant dissimilarity between optimized SRP formulation and reference sample. In addition, SRP is soluble in water; therefore, in order to prevent it from hydrolysis during the preparation of enteric-coated tablets, it is suggested to adopt dry granulation or ethanol granulation method.

#### 4.2.7. Determination of Assay

**Figure 9:**
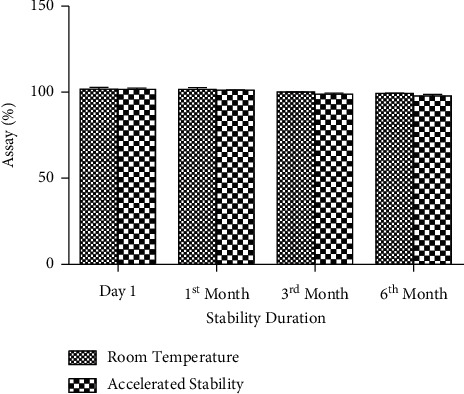
Comparative assay of SRP between room temperature and accelerated stability.

The assay percentage of SRP was analyzed as per the procedure mentioned in Section 2.3.4. During day 1, the calculated assay for room temperature and accelerated analysis were 101.82 and 101.64%, respectively. Until 6 months of stability, both room temperature and accelerated stability analysis exhibited assay % within the limit. There was no significant degradation in assay throughout the stability period. The corresponding figures for room temperature and accelerated stability were 99.32 and 97.91%, respectively, in 6th month's analysis. The overall mean RSD % was 1.51%. The detailed observations of assay are demonstrated in Figure 9.

## 5. Conclusions

In this study, enteric-coated tablet formulation of SRP was prepared and a simple validated UV method was developed for analytical determination and quantification of SRP in formulated tablets. The drug release profile of our formulated tablet at phosphate buffer (pH 6.8) and simulated intestinal fluid (pH 6.8) was relatively higher than the marketed sample of SRP tablet. The results of initial and accelerated stability until 6 months revealed that the formulated tablets were both physically and chemically stable. However, still adequate studies based on biological stability and hydrolysis are suggested to verify the stability and efficacy. From this study, it was concluded that, by employing commonly available pharmaceutical excipients such as diluents, superdisintegrants, and hydrophilic and hydrophobic polymers, a commercial enteric-coated tablet of SRP could be developed successfully. This study can contribute as a guide to the pharmaceutical companies to manufacture SRP containing dosage forms and apply validated analytical methods to assess its product quality.

## Figures and Tables

**Table 1:** Composition of formulated tablets.

S. No.	Materials (mg/tablet)	*F*1	*F*2	*F*3	*F*4	*F*5
1.	Serratiopeptidase	10.50	10.50	10.50	10.50	10.50
2.	MCCP pH 101	141.00	—	—	—	—
3.	MCCP pH 102	—	139.50	132.50	132.50	132.50
4.	Colloidal silicon dioxide	2.00	2.50	4.00	4.00	4.00
5.	Sodium starch glycolate	3.50	4.50	5.50	5.50	5.50
6.	Crospovidone	—	—	4.00	4.00	4.00
7.	Magnesium stearate	1.50	1.50	2.00	2.00	2.00
8.	Stearic acid	1.50	1.50	1.50	1.50	1.50
Total weight (uncoated)		160.00	160.00	160.00	160.00	160.00

Seal coating materials						
1.	White shellac	—	—	5.10	5.70	6.37
2.	Castor oil	—	—	0.062	0.062	0.062
3.	Isopropyl alcohol	—	—	105.00	105.00	105.00

Enteric coating materials						
1.	HPMC phthalate 40	—	—	8.00	8.00	13.44
2.	Ethyl cellulose	—	—	2.74	2.74	3.820
3.	Castor oil	—	—	0.47	0.47	0.97
4.	Titanium dioxide	—	—	0.55	0.55	0.55
5.	Lake Erythrosine	—	—	0.11	0.11	0.11
6.	Isopropyl alcohol	—	—	85.00	85.00	85.00
7.	Methylene chloride	—	—	150.00	150.00	150.00
Targeted weight in mg (coated)		—	—	170.00	170.00	170.00

**Table 2:** Accuracy analysis of SRP.

Preanalyzed concentration of serratiopeptidase: 50 *µ*g/mL									
Level (%)	Concn. of std. added (*µ*g/mL)	Total concn. (*µ*g/mL)	Abs.	Avg. abs.	Concn. found (*µ*g/mL)	Recovery %	RSD %	Mean	SD
50	25	75	0.129	0.131	74.9	99.9	0.191	100.14	0.192
	25	75	0.131						
	25	75	0.134						

100	50	100	0.191	0.191	100.3	100.3	0.190	99.98	0.190
	50	100	0.188						
	50	100	0.194						

150	75	125	0.238	0.241	125.2	100.2	0.190	100.36	0.191
	75	125	0.241						
	75	125	0.244						

**Table 3:** Intraday analysis of SRP.

Average (*n* = 3) SD, RSD %	50 (*µ*g/mL)	100 (*µ*g/mL)	150 (*µ*g/mL)
	0.10137	0.19507	0.28463
	0.00014	0.00031	0.00027
	0.14	0.16	0.10

**Table 4:** Interday analysis of SRP (analyst 1).

Average (*n* = 3) SD, RSD %	50 (*µ*g/mL)	100 (*µ*g/mL)	150 (*µ*g/mL)
	0.10115	0.19502	0.28442
	0.00017	0.00022	0.00024
	0.17	0.11	0.08

**Table 5:** Interday analysis of SRP (analyst 2).

Average (*n* = 3) SD, RSD %	50 (*µ*g/mL)	100 (*µ*g/mL)	150 (*µ*g/mL)
	0.10111	0.19497	0.28438
	0.00015	0.00017	0.00017
	0.15	0.09	0.0006

**Table 6:** LOD and LOQ of SRP.

Parameters	Data
Slope (*n* = 6)	0.001
SD (*n* = 6)	0.0016
LOD (*µ*g/mL)	5.28
LOQ (*µ*g/mL)	16.00

**Table 7:** Robustness data of SRP.

Concentration (*µ*g/mL)	Absorbance at 264.7	Absorbance at 265.7	Absorbance at 266.7
100 (*n* = 3)	0.1862	0.1874	0.1934
100 (*n* = 3)	0.1814	0.1824	0.1883
100 (*n* = 3)	0.1775	0.1831	0.1898
Mean	0.1817	0.1843	0.1905
SD	0.00316	0.002	0.002
RSD %	1.73	1.08	1.04

**Table 8:** LOD and Hausner ratio of granules.

Formulations	LOD	Hausner ratio
*F*1	2.28	1.25
*F*2	2.37	1.17
*F*3	2.25	1.22
*F*4	1.72	1.052
*F*5	1.83	1.09

**Table 9:** Assessment of weight variation, thickness, and diameter.

Tablet specification	Average	Minimum	Maximum
Weight variation (mg), *n* = 20	172.77	171.50	178.10
Thickness (mm), *n* = 10	3.94	3.92	3.97
Diameter (mm), *n* = 10	7.42	7.41	7.44

**Table 10:** Comparative evaluation between room temperature and accelerated stability.

Duration	Temp. (ºC)	Humidity (%)	^a^Hardness (kgf)	Color change
1 day	25	NA	23.02 ± 0.17	—
	40	75	22.87 ± 0.12	—

1 month	25	NA	22.45 ± 0.24	—
	40	75	21.68 ± 0.19	—

3 months	25	NA	21.12 ± 0.11	—
	40	75	20.54 ± 0.22	—

6 months	25	NA	20.55 ± 0.17^*∗*^	—
	40	75	19.37 ± 0.08^*∗*^	—

^a^Values are expressed as mean ± SD (*n* = 6); −: absence; NA: not applicable. ^*∗*^*p* < 0.05 compared to day 1.

**Table 11:** The percentage of the residual content of SRP enteric-coated tablets after agitation in simulated gastric fluid (pH 1.2) for 2 h.

Samples tested	Times (%)						Average (%)
	1	2	3	4	5	6	
SRP formulated tablets	98.40	99.14	98.35	99.24	98.12	97.69	98.49
Market sample	98.22	97.10	96.42	94.14	96.29	95.42	96.27

## Data Availability

No external data were used to support this study.
